# Erythropoietin Couples Hematopoiesis with Bone Formation

**DOI:** 10.1371/journal.pone.0010853

**Published:** 2010-05-27

**Authors:** Yusuke Shiozawa, Younghun Jung, Anne M. Ziegler, Elisabeth A. Pedersen, Jianhua Wang, Zhuo Wang, Junhui Song, Jingcheng Wang, Clara H. Lee, Sudha Sud, Kenneth J. Pienta, Paul H. Krebsbach, Russell S. Taichman

**Affiliations:** 1 Department of Periodontics and Oral Medicine, University of Michigan School of Dentistry, Ann Arbor, Michigan, United States of America; 2 Department of Biologic and Materials Sciences, University of Michigan School of Dentistry, Ann Arbor, Michigan, United States of America; 3 Shanghai Jiao-Tong University School of Medicine, Institutes of Medical Sciences, Shanghai, People's Republic of China; 4 Departments of Internal Medicine and Urology, University of Michigan School of Medicine, Ann Arbor, Michigan, United States of America; Universidade do Porto, Portugal

## Abstract

**Background:**

It is well established that bleeding activates the hematopoietic system to regenerate the loss of mature blood elements. We have shown that hematopoietic stem cells (**HSCs**) isolated from animals challenged with an acute bleed regulate osteoblast differentiation from marrow stromal cells. This suggests that HSCs participate in bone formation where the molecular basis for this activity is the production of BMP2 and BMP6 by HSCs. Yet, what stimulates HSCs to produce BMPs is unclear.

**Methodology/Principal Findings:**

In this study, we demonstrate that erythropoietin (**Epo**) activates Jak-Stat signaling pathways in HSCs which leads to the production of BMPs. Critically, Epo also directly activates mesenchymal cells to form osteoblasts *in vitro*, which *in vivo* leads to bone formation. Importantly, Epo first activates osteoclastogenesis which is later followed by osteoblastogenesis that is induced by either Epo directly or the expression of BMPs by HSCs to form bone.

**Conclusions/Significance:**

These data for the first time demonstrate that Epo regulates the formation of bone by both direct and indirect pathways, and further demonstrates the exquisite coupling between hematopoesis and osteopoiesis in the marrow.

## Introduction

Osteoblasts play a central role in skeletal development. Derived from pluripotent mesenchymal stem cells (**MSCs**), osteoblasts mature along a specific lineage to become highly specialized synthetic cells. As such, osteoblasts respond to many mechanical, local and systemic stimuli that regulate mineralization while orchestrating bone remodeling. Work by our group in the human system and others in murine models have demonstrated that osteoblasts also constitute part of the stromal cell support system in marrow for hematopoiesis by participating in the formation of the HSC niche (reviewed in [1]). In this capacity osteoblast-expressed cell-to-cell receptors (e.g., N-Cadherin, Jagged, VCAM-1, Annexin II), soluble and cell-surface associated cytokines, and growth factors all regulate HSC functions. Each of these factors are in turn influenced by hormonal (e.g., PTH) and local signals (e.g., BMPs, Ang-1) [1], [2].

It is believed that cross talk between hematopoietic cells and bone forming osteoblasts regulates each other's function [3], [4]. In fact, as late as the 19th century bloodletting was a frequent medical procedure for a number of conditions including the treatment of metabolic bone diseases [5], [6]. Since the mid 1990's, however, it has been known that direct marrow ablation, or trauma is potent activators of osteogenic activities *in vivo* [7], [8], [9], [10], [11], [12]. It has also been demonstrated that blood loss, a condition that stimulates hematopoietic stem cells (**HSCs**), may also activate osteoprogenitor cells in the bone marrow to form bone [12]. Animals that are subjected to an acute blood loss (1% or 3% of body weight) had significant increases in mineral appositional rates, osteoblast number, and serum levels of an osteogenic growth peptide [12]. These findings suggest that bleeding alone, without skeletal trauma, may induce an osteogenic response that is systemic in nature. Yet, the mechanisms induced by direct marrow injury and bleeding to stimulate bone formation remains unclear [12].

We have also recently shown that HSCs isolated from animals challenged with an acute bleed regulate osteoblast differentiation from mixed bone marrow stromal cells (**BMSCs**) and MSCs, suggesting that HSCs actively participate in bone formation [2]. The molecular basis for this activity is the production of BMP2 and BMP6 by HSCs [2]. These findings support the reports that show that an acute bleed activates bone formation *in vivo* [7], [8], [9], [10], [11], [12]. Yet, what stimulates HSCs to secrete BMPs is unknown. We hypothesized that the factor that stimulates HSCs to secrete BMPs and ultimately bone formation was erythropoietin (**Epo**). Although Epo is best known for its role as a hematopoietic hormone, recent findings that Epo receptors (**EpoR**) are expressed in non-hematopoietic tissues (endothelial cells, neurons, trophoblast cells) has hastened the search for non-hematopoietic effects of Epo [13], [14], [15], [16], [17]. Yet there are no examples in the literature that directly link Epo to bone formation. In present study, we show that Epo activates HSCs to produce BMPs. In addition, it was found that Epo acts directly on BMSCs to induce osteoblastic differentiation. Importantly, Epo stimulates bone formation *in vivo* by targeting both HSCs and the osteoblastic niche. These findings demonstrate that Epo directly and indirectly regulates the formation of bone, and further demonstrates an exquisite coupling between hematopoiesis and osteopoiesis in the marrow.

## Results

### Epo stimulates BMP expression by HSCs

**Figure 1:**
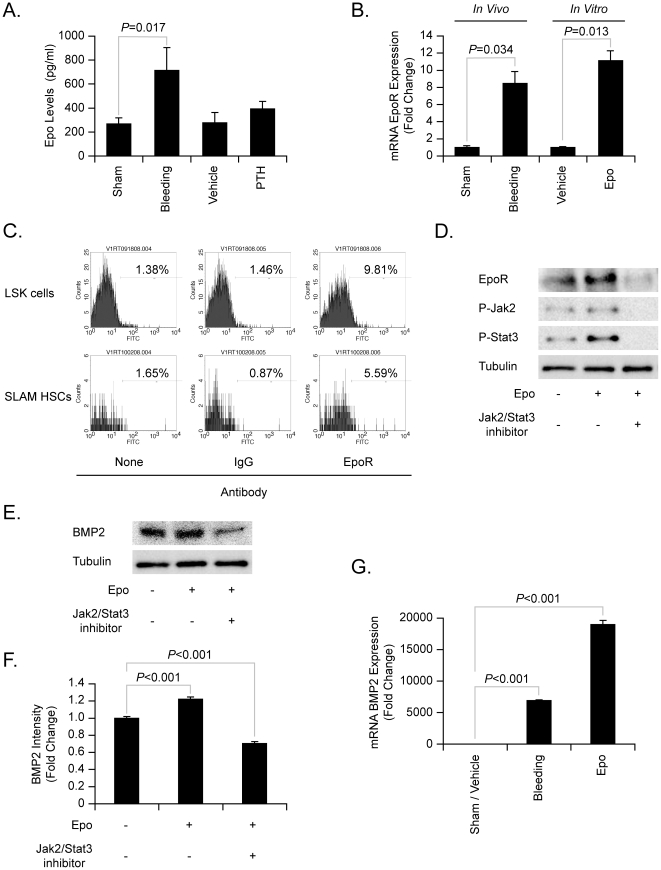
HSCs express EpoR and Epo stimulates HSCs to express BMPs. (**A**) Serum Epo levels in sham (non-bled), bled animals, vehicle-treated animals, or PTH treated animals (n = 5 per group) were determined by ELISA. Data are presented as the mean ± standard error of the mean. Significant difference from the sham bled animals. (**B**) Real-time RT-PCR detection of EpoR mRNA levels in HSCs isolated using SLAM-family receptors. *In vivo*: mRNA recovered from HSCs isolated directly from sham bled or animals bled 2 days prior. *In vitro*: mRNA recovered from naïve HSCs treated with vehicle or rhEpo (10 U/ml) for 12 hours. The data were normalized to the expression of β-Actin. Data from a representative of three experiments are presented as the mean ± standard error of the mean. Significant differences from the sham bled animals or vehicle treatment groups. (**C**) FACS analysis for EpoR expression by HSCs isolated using LSK or SLAM-family markers. (**D**) Western blot analysis for the expression of EpoR, P-Jak2, P-Stat3 expression by HSCs (LSK markers) treated with Epo with or without AG490 (an inhibitor of Stat3 and Jak2) for 12 h. P: phosphorylated. (**E**) Western blot analysis for BMP2 expression by HSCs (LSK markers) treated with Epo with or without AG490 after 12 h, (**F**) Quantitative digital image analyses of BMP2 expression. Data from a representative of three experiments are presented as the mean ± standard error of the mean. Significant differences from vehicle treatment groups. (**G**) Real-time RT-PCR detection of BMP2 mRNA levels expressed by HSCs (SLAM markers) following sham bleeding/vehicle treatment, bleeding, or *in vivo* Epo (6000 U/kg) stimulation, 2 days prior to sacrifice (n = 5 per group). Data are presented as the mean ± standard error of the mean. Significant differences from sham bleeding/vehicle treatment groups.

We have demonstrated that HSCs respond to an acute bleed by secreting BMPs, which are able to stimulate MSC differentiation to osteoblastic lineage *in vitro* and *in vivo* [2]. What activates HSCs to secrete BMPs is however unknown. We hypothesized that Epo may serve as the link between bleeding and BMPs production by HSCs. Therefore, to address the hypothesis, it was first determined if the serum levels of Epo are able to increase during the same time frame that HSC activation occurs. For these studies, animals were bled and 3 days later serum was collected. As expected, serum Epo levels rose in the bled animals (**Figure 1A**), whereas PTH, a hormone known to stimulate bone formation [18], failed to enhance the serum Epo levels (**Figure 1A**).

Epo is known to signal through the Epo receptor, a member of the hematopoietic/cytokine/growth factor receptor family [19]. A comparison of the expression of mRNA for the EpoR in HSCs (isolated by means of the SLAM-family of receptors) demonstrated that bleeding increased the mRNA levels for the EpoR (**Figure 1B** **left**). Intriguingly, when HSCs were isolated from naïve animals and treated with Epo *in vitro*, a similar increase in EpoR expression was observed (**Figure 1B** **right**). Further evaluation of the expression of the EpoR at the protein level was performed by flow cytometry using Alexa488-conjugated anti-EpoR antibodies. Approximately 10% of the HSCs isolated on the basis of Lin^−^Sca1^+^cKit^+^ (**LSK**) markers or 5% of HSCs isolated using the SLAM-family of receptors express EpoR under basal conditions (**Figure 1C**), suggesting that activation of resting HSCs by Epo is possible.

To determine if Epo is able to signal through EpoR in HSCs, cells were cultured for 12h *in vitro* in the presence or absence of Epo [20]. *In vitro*, Epo stimulation increased the expression of EpoR and resulted in the phosphorylation of Jak2 and Stat3 in HSCs (isolated using LSK markers) (**Figure 1D**, quantified in **Figure S1A-C**). Inclusion of the Jak2 and Stat3 phoshorylation inhibitor AG490 (Calbiochem, La Jolla, CA) into the culture prevented Epo activation of Jak2 and Stat3 (**Figure 1D**, quantified in **Figure S1A-C**). Most importantly, when Epo is used to stimulate HSCs *in vitro*, more BMP2 mRNA was observed (**Figure 1E**, quantified in **Figure1F**). To confirm that the *in vitro* observations are relevant *in vivo*, the BMP2 levels in HSCs (isolated using the SLAM-family receptors) were examined in HSCs recovered from the Epo-treated (6000 U/kg) or vehicle-treated animals. When the animals were bled, Epo significantly stimulated BMP levels in HSCs (**Figure 1G**), as seen before [2]. These data suggest that HSCs express EpoR on their surfaces, and that Epo induces BMP2 expression by HSC through the Jak2/Stat3 signaling pathways.

### Epo acts directly on bone marrow stromal cells to induce an osteoblastic phenotype

**Figure 2:**
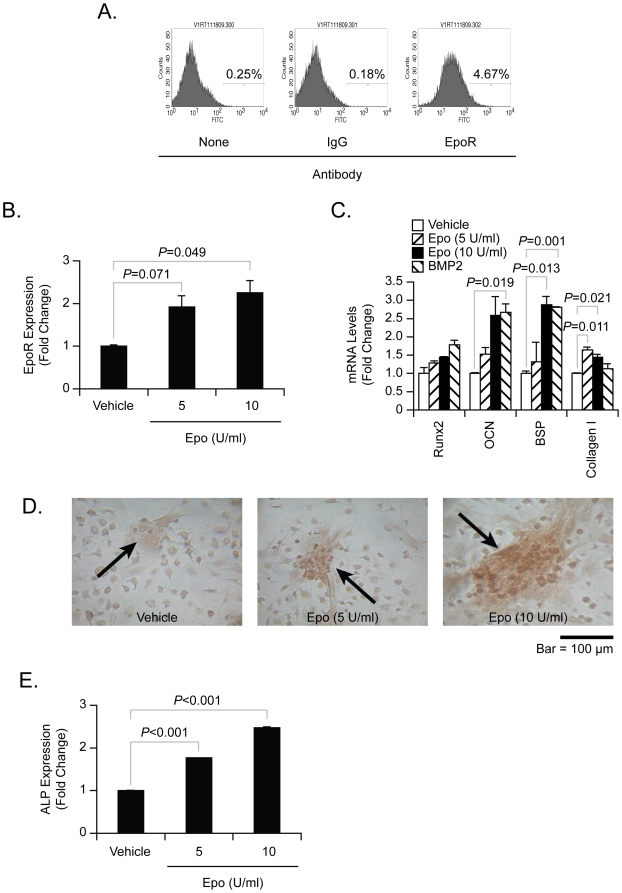
Mixed bone marrow stromal cells (BMSCs) are induced towards an osteoblastic phenotype by Epo. (**A**) FACS analysis for EpoR expression by BMSCs. (**B**) Real-time RT-PCR determination for the expression of EpoR mRNA by BMSCs following vehicle or Epo treatment. The data were normalized to β-Actin. Data from a representative of three experiments are presented as the mean ± standard error of the mean. Significant difference from vehicle treatment. (**C–E**) BMSCs were cultured for 21 days in mineralizing conditions (ascorbic acid & β-glycerol phosphate) in the presence or absence of Epo (5–10 U/ml) and BMP2 (200 ng/ml; as a positive control). (**C**) The expression of Runx2, OCN, BSP, Collagen I mRNA were detected by real-time RT-PCR. (**D**) In vitro mineralization in response to Epo was demonstrated by Alizarin red staining. Original magnification at 40×. Bar = 100 microns. (**E**) Alkaline phosphatase activity measured in BMSC lysates following Epo treatment. Data from a representative of three experiments are presented as the mean ± standard error of the mean. Significant differences from vehicle treatment groups.

Recent findings suggest that non-hematopoietic cells express EpoR, and therefore non-hematopoietic effects of Epo are possible [21]. Next, it was determined if circulating Epo directly targets stromal cells in the marrow to activate osteoblastic activities. To determine if bone marrow stromal cells (BMSCs) express EpoR, EpoR protein levels were first evaluated by flow cytometry (**Figure 2A**). As shown **Figure 2B**, EpoR mRNA increases following Epo Treatment. To evaluate if Epo stimulates osteoblastic differentiation, BMSCs were cultured for 21 days in mineralizing conditions (ascorbic acid & β-glycerol phosphate) in the presence or absence of Epo and BMP2 (as a positive control). As expected, BMP2 stimulated osteoblastic differentiation (**Figure 2C**), including induction of the bone specific transcription factor Runx2, osteocalcin (**OCN**) and bone sialoprotein (**BSP**). Epo treatment of the culture similarly induced the expression of a bone phenotype including induction of Runx2, OCN and BSP (**Figure 2C**). In addition, Epo was able to increase mineral deposition by the cultures (**Figure 2D**) and enhanced the expression of Alkaline phosphatase (**ALP**) activity (**Figure 2E**). These data suggest that BMSCs express EpoR, and that Epo directly induces osteoblastic differentiation of BMSCs.

### Epo induces osteoclastogenesis but not osteoclast function

**Figure 3:**
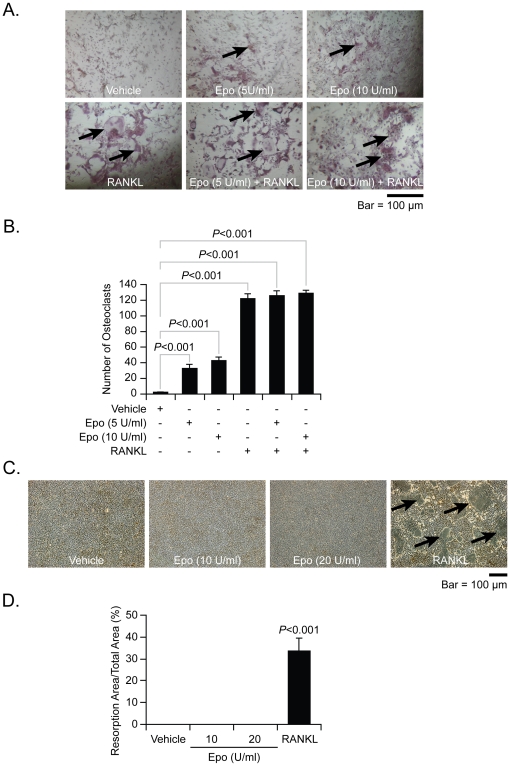
Epo supports osteoclastogenesis but not bone resorbing activities. To evaluate if Epo regulates osteoclastogenesis, marrow mononuclear cells (**MMCs**) (1×10^5^ cells/well) were treated with rhEpo (5–10 U/ml) and/or RANKL (50 ng/ml (as a postitive control) in 96-well plates. At 5 days, staining for TRAP was performed. (**A**) Representative light-microscopic images or cells expressing TRAP in mixed MMC culture. Original magnification at 40×. Bar = 100 microns. (**B**) The numbers of TRAP-positive cells from quantified in (A). Data from a representative of three experiments are presented as the mean ± standard error of the mean. Significant differences from vehicle treatment groups. To examine if Epo stimulate the function of osteoclasts, MMCs (5×10^5^ cells/well) were seeded on the resorbable artificial bone film-coated disks and incubated with rhEpo (10–20 U/ml), or rhRANKL (50 ng/ml). (**C**) Representative micrographs demonstrating bone resorption and resorption area quantified in (**D**). Original magnification at 20×. Bar = 100 microns. Data from a representative of three experiments are presented as the mean ± standard error of the mean. Significant differences from vehicle treatment groups.

Since osteogenesis and osteoclastogenesis are functionally linked, it was next determined if Epo directly regulates osteoclastogenesis. For these studies, marrow mononuclear cells (**MMCs**) were treated with Epo for 5 days. Receptor activator of NFκβ (**RANKL**) was included in the assays as a positive control of osteoclastogenesis. At the conclusion of the studies, TRAP staining was performed and multinucleated TRAP-positive cells in the cultures were counted. As expected, the RANKL enhanced osteoclastogenesis in MMCs (**Figure 3A**, quantified in **Figure 3B**). Compared to the vehicle treatments, Epo stimulated osteoclast formation by MMCs (**Figure 3A**, quantified in **Figure 3B**). Next, to determine if Epo activates osteoclastic activity, *in vitro* bone resorption assays were performed. Intriguingly, Epo had no effects on bone resorption induced by osteoclasts, while RANKL induced bone resorption (**Figure 3C**, quantified in **Figure 3D**). These data suggest that Epo induces osteoclast formation, but alone does not stimulate osteoclastic function in the marrow.

### Epo stimulates bone formation *in vivo*

**Figure 4:**
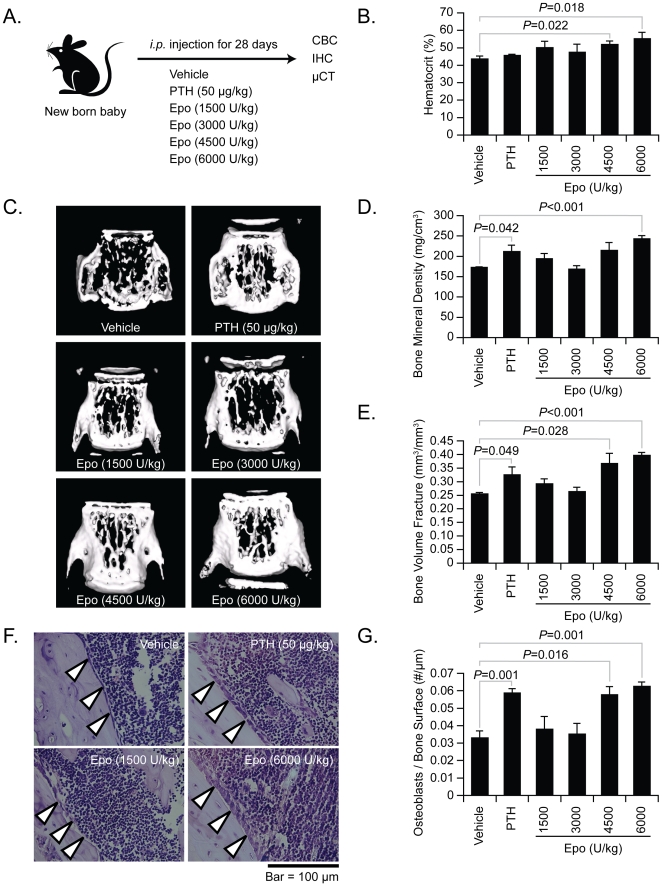
Epo induces bone formation *in vivo* in neonatal animals. (**A**) Experimental model. To determine if Epo is able to stimulate bone formation *in vivo*, newborn mice were injected with vehicle or increasing concentrations of Epo (1500–6000 U/kg, 3 times/week) for 28 days *i.p.* As a positive control for bone formation, PTH was administered using an anabolic regime (50 µg/kg *s.c.*, daily). n = 5 per group. (**B**) Epo increases hematocrit levels over the course of the 28 day experiment. (**C**) Micro-computed tomography measurements of the vertebral spines were performed on fixed vertebral bodies at the conclusion of the study. Bone parameters including (**D**) BMD and (**E**) BVF were calculated from the micro-computed tomography measurements. (**F**) Representative H&E staining of the long bones of treated animals. (**G**) Osteoblasts numbers were quantified on the long bone sections with H&E staining. Original magnification at 60×. Bar = 100 microns. Data are presented as the mean ± standard error of the mean. Significant differences from vehicle treatment groups.

To directly assess the ability of Epo to stimulate bone formation *in vivo*, new born mice were treated with vehicle or increasing concentrations of Epo (1500–6000 U/kg, 3 times/week) for 28 days (**Figure 4A**). As a positive control for bone formation PTH was administered (50 µg/kg, daily) [22]. Compared to control vehicle treatments, Epo significantly increased the hematocrit (**Figure 4B**), hemoglobin and red blood cell counts in blood of all treated animals (**Data not shown**). Whereas PTH did not have any discernable effects on any of the blood parameters examined, nor did PTH alter serum Epo levels (**Data not shown**). Critically, micro-computed tomography (**μCT**) of the vertebral spines demonstrated that like PTH treatments, Epo significantly increased bone mineral density (**BMD**) and bone volume fraction (**BVF**), compared to vehicle control (**Figure 4C**, and quantified in **Figure 4D&E**). Histomorphometric analyses of the long bones also demonstrated that Epo increased the number of osteoblasts on the bone surfaces of the treated animals compared to controls (**Figure 4F**, quantified in **Figure 4G**).

**Figure 5:**
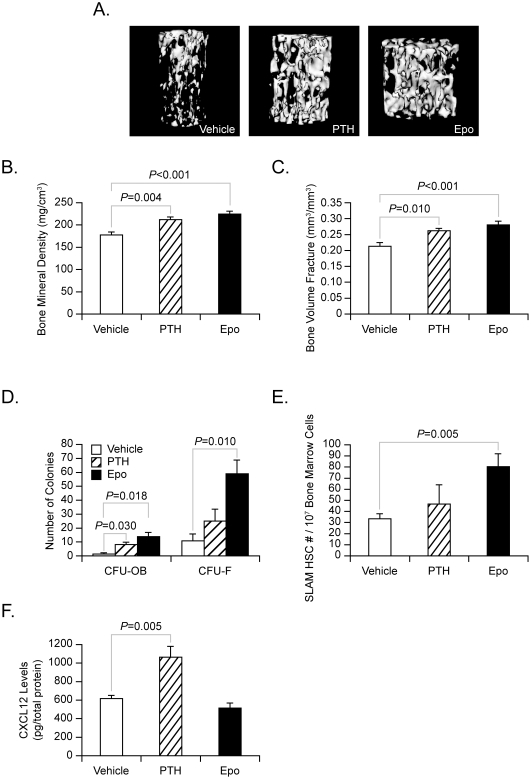
Epo induces bone formation *in vivo* in mature animals. To determine if Epo is able to stimulate bone formation in mature animals, 4-6-week-old mice were injected with vehicle or increasing concentrations of Epo (6000 U/kg, 3 times/week) for 28 days *i.p.* As a positive control for bone formation, PTH was administered using an anabolic regime (50 µg/kg *s.c.*, daily). n = 5 per group. (**A**) The vertebral spines were fixed and 3-dimensional micro-computed tomography measurements of the vertebral spines were performed. Bone parameters including (**B**) BMD and (**C**) BVF were calculated. (**D**) Mixed marrow mononuclear cells derived from the treated-animals were cultured in mineralizing conditions (ascorbic acid & β-glycerol phosphate). At 21 days, the cultures were examined for CFU-OB (Alizarin Red stainig) or CFU-F (Crystal Violet staining) colonies. (**E**) The numbers of SLAM HSCs were counted by FACS. (**F**) The levels of CXCL12 in the marrow were measured by ELISA. Data are presented as the mean ± standard error of the mean. Significant differences from vehicle treatment groups.

To determine if Epo is able to regulate bone formation in mature animals, the animal studies were repeated by starting the Epo treatments when the animals were 4–6 weeks of age. For these studies, 6000 U/kg of Epo was chosen as treatment dose, since this dose showed significant skeletal effects on new born mice. Like those shown in Figure 4, both BMD and BVF levels were enhanced by Epo treatment in mature animals (**Figure 5A**, and quantified in **Figure 5B&C**). To determine if Epo stimulated induces MSCs differentiation along the osteoblastic lineage *in vivo*, osteoblast colony-forming unit (**CFU-OB**) and fibroblast colony-forming unit (**CFU-F**) assays were performed on marrow recovered from the treated animals. Interestingly, Epo treatments were significantly better able to induce CFU-OB and CFU-F differentiation compared to the vehicle treatment (**Figure 5D**).

In previous work it has been demonstrated that HSC numbers correlate with increases in the osteoblast numbers in marrow [23]. To determine if increases in HSC numbers accompanies an increase in osteoblastic parameters, the numbers of HSC were evaluated by FACS. Significantly, more HSCs (isolated using the SLAM-family receptors) were found in the marrow from Epo-treated animals (**Figure 5E**). Previously, it has been observed that expansion of HSC pools is regulated in part by changes in marrow levels of stromal derived factor-1 (SDF-1 or CXCL12). Accordingly, CXCL12 levels in the marrow were evaluated by ELISA. Surprisingly, Epo treatment did not enhance the CXCL12 levels in the marrow, while PTH did (**Figure 5F**). These data suggest that Epo treatment stimulates the bone formation in both neonatal and mature animals *in vivo*, and further suggest that at least two independent pathways to support the bone development. One pathway is regulated via PTH. The other is regulated by Epo.

**Figure 6:**
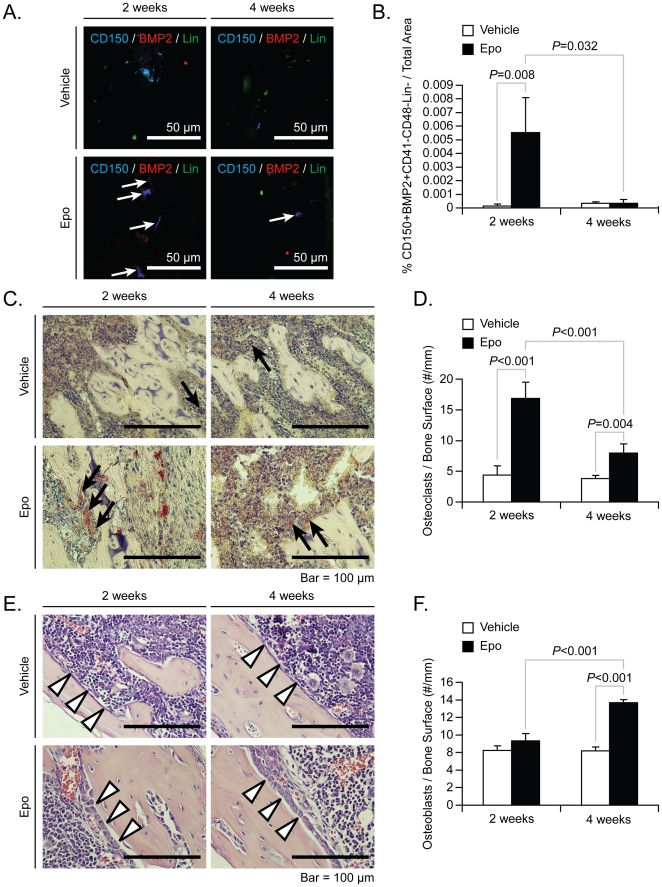
Epo regulates the balance between the osteoblastogenesis and the osteoclastogenesis. To determine if Epo is able to stimulate bone formation in mature animals, 4-6-week-old mice were injected with vehicle or increasing concentrations of Epo (6000 U/kg, 3 times/week) for 28 days *i.p.* n = 5 per group. At 2 weeks and 4 weeks, animals were sacrificed and the long bones were prepared for immunohistochmistry. (**A**) Representative elements of the marrow triple-stained with anti-CD150 antibodies, anti-BMP2 antibodies, and anti-lineage antibody cocktail. (**B**) CD150^+^BMP2^+^CD41^−^CD48^−^Lin^−^ area were quantified on the long bone sections. Original magnification at 60×. Scale Bar = 50 microns. (**C**) Representative TRAP staining of the long bones of treated animals. (**D**) Osteoclasts numbers were quantified on the long bone sections with Trap staining. Original magnification at 60×. Bar = 100 microns. (**E**) Representative H&E staining of the long bones of treated animals. (**F**) Osteoblasts numbers were quantified on the long bone sections with H&E staining. Original magnification at 60×. Bar = 100 microns. Data are presented as the mean ± standard error of the mean.

Bone formation depends upon the balance between the osteoblastogenesis and the osteoclastogenesis. We have shown that Epo stimulates osteoclast and osteoblast formation, and activates HSCs. To determine the temporal relationships between these events, osteoblast and osteoclast numbers were evaluated over time. The levels of BMP2 mRNA expresed by HSCs were significantly elevated in Epo-treated animals by two weeks compared to vehicle-treated animals. However, the levels of BMP2 expression expressed by HSCs returned near to base line by 4 weeks. Immunohistochemistry was used to confirm the BMP2 mRNA data. By staining for HSCs in the marrow (CD150^+^CD41^−^CD48^−^Lin^−^ cells), it was noted that the number of HSCs that express BMP2 was increased in the marrow at 2 weeks of Epo treatment (**Figure 6A**, and quantified in **Figure 6B**). But by four weeks, the number of HSCs expressing BMP2 had decreased. Epo stimulated formation of osteoclasts by 2 weeks, but by 4 weeks, their numbers had begun to return to baseline levels (**Figure 6C**, and quantified in **Figure 6D**). As expected, while there were no remarkable differences in osteoblast numbers between vehicle and Epo treatment at 2 weeks. However, by 4 weeks of treatment, Epo significantly increased the number of osteoblasts observed *in vivo* (**Figure 6E**, and quantified in **Figure 6F**). These data suggest that Epo first activates osteoclastogenesis which is later followed by osteoblastogenesis either directly or by the expression of BMPs by HSCs to induce bone formation.

## Discussion

It is well established that bleeding activates the hematopoietic system to regenerate the loss of mature blood elements. To accommodate the space required for this activity in marrow, bone is resorbed. Yet, it was not clear how the lost bone is repaired, when a hematopoietic steady state is re-established.

**Figure 7:**
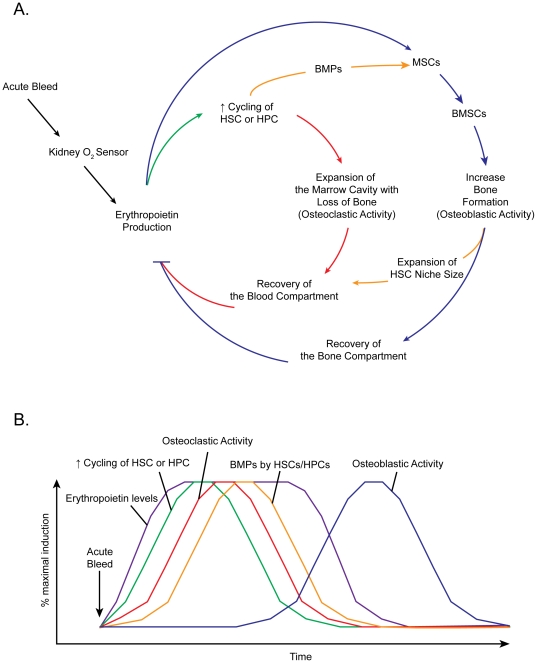
Coupling of hematopoiesis with osteopoiesis by Epo. Erythropoietin (**Epo**) is best known as a hematopoietic hormone. Epo is now known to have a number of non-hematpoietic roles. However, what is not clear is how the bone compartment recovers. (**A**) Model of hematopoetic/osteopoiesis coupling by Epo. An acute bleed activates the kidney O_2_ sensors to secrete Epo. In the marrow, Epo activates hematopoietic stem cell (**HSC**) or progenitor cell (**HPC**) to enter the cycle (**Green line**), which results in the expansion of hematopoietic organ at the expense of the bone tissue by activating osteoclastogenesis. Ultimately, expansion of the hematopoietic organ leads to the restoration of the formed elements of the blood (**Red lines**). At the same time, Epo acts directly on mesenchymal stem cells (**MSCs**) and mixed bone marrow stromal cells (**BMSCs**) to induce bone formation. Ultimately, activation of MSCs and BMSCs results in the restoration of the bone tissues (**Blue lines**). In addition to the direct effects of Epo on HSCs, HPCs, and BMSCs, Epo activates HSCs and HPCs to secrete BMPs that target MSCs/BMSCs to reprogram the cells to differentiate into osteoblasts to regenerate the lost bone tissue. Moreover, the osteoblasts that are induced by Epo directly or indirectly support hematopoiesis as a bone marrow niche (**Orange lines**). (**B**) Time plot graph of events activated by Epo. Elevation of circulating Epo levels (**Purple line**) facilitates expansion of the HSCs/HPCs to restore formed blood elements lost during an acute bleed (**Green line**), and to activate osteoclastogensis to expand the marrow cavity (**Red line**). At the same time Epo induces BMPs expression by HSCs/HPCs to promote bone formation indirectly (**Orange line**). At a later time, osteoblast number increases in the marrow stimulated directly by Epo's actions on MSCs/BMSCs and indirectly by BMPs secreted by HSCs/HPCs (**Blue line**). Together, Epo plays a critical and important role in cross talk between hematopoiesis and osteopoiesis by both direct and indirect pathways.

We previously demonstrated that HSCs regulate the development of their own niche by producing of BMPs [2]. In a search for the activity that stimulates HSCs to produce BMPs, we hypothesized that the factor was Epo. Our data demonstrates that a functional EpoR is expressed by HSCs, and that Epo signaling leads to BMP production by HSCs. We also found that Epo is able to stimulate BMSCs to form bone tissue directly. While the precise cellular target in the mixed bone marrow stromal population remains unclear, preliminary PCR results suggest that very small cells with embryonic features (CD45^−^Sca-1^+^Lin^−^ cells) express that are capable of multi-lineage differentiation express EpoR (manuscript in preparation). Thus, by either activating HSCs or BMSCs, Epo couples hematopoiesis with bone formation (see model, **Figure 7A&B**).

Recently several groups have demonstrated that events centered at the endosteal surfaces are critical for hematopoietic regulation particularly by cells which share an osteoblastic phenotype in mouse models [23], [24]. These studies extend our work using human cells in which we have shown that soluble signals secreted by HSC establish a paracrine loop with osteoblasts that supports early stem cell survival and suggest that HSCs modulate the formation of a hematopoietic microenvironment directly. The studies reported here are also in line with preclinical evidence in rodents suggests that bleeding, in addition to stimulating hematopoiesis, stimulates bone formation [7], [8], [9], [10], [11], [12]. For example, acute blood loss increases the rate of reparative bone regeneration in animals [25]. However, most of the investigation in this area has focused on what happens to bone under states of hematopoietic stress. The enhanced production of hematopoietic marrow has long been known to be associated with the conversion of fatty marrow to hematopoietic marrow at the expense of bone. This occurs particularly in the appendicular skeleton, and is associated with accelerated bone resorption observed in chronically bled animals. Similar findings may be responsible for progression of osteoporosis in postmenopausal women where chronic menstrual bleeding may persist for up to 35 years [26]. Where it has been estimated that 70 ml of blood is lost monthly for a total of ∼850 ml/year and nearly 30 L over a life time [26]. Likewise, osteoporosis is a common finding in β-thalassemia, and bone mineral densities are improved in transfused individuals [27]. On the other hand, an acute change in altitude, and by inference Epo, may increase MSC numbers in patients with cytolytic syndromes [28]. Here, it was noted that fibroblastic colony-formation was 2 to 4 times increased after altitude change of 3,200 m [28].

Yet, direct evidence that Epo stimulates osteopoiesis or bone formation is scarce and reports are often at odds with each other. For example, it was recently demonstrated that Epo treatment in a murine closed femur fracture model significantly improved healing during early facture repair (days 0–6), but had little long term effects [16]. These data suggest that Epo may induce BMSC differentiation towards an osteoblastic phenotype. Conversely, the presence of a developing callus after fracture inhibits erythropoiesis which could explain a mechanism for the recovery of the hematopoietic system following marrow expansion [29]. Recent work by Yamaza et al suggests that part of the mechanism involved in the organizing of bone/hematopoietic marrow organ is that EpoR-positive stromal cells may represent a small subset of the heterogeneous population of the bone marrow [30]. Recently, a condition described as adynamic bone disease which is characterized by a defect in bone matrix formation and mineralization in uremic patients has been defined by double labeled bone biopsy specimens. While the pathogenesis of adynamic bone disease is unclear, one hypothesis that has been put forth is that Epo administration is associated with increased indexes of bone matrix formation [31]. On the other side, the effects of Epo on bone metabolism in patients receiving chronic hemodialysis were directly assessed [31]. The results show that exogenous Epo administration correlated with the increases in 1,25-dihydroxy-vitamin D (r = 0.38, p<0.05) serum levels, intact osteocalcin (r = 0.42, p<0.05) and bone alkaline phosphatase (r = 0.53, p<0.005), but not intact parathyroid hormone (r = 0.09) [31]. The findings raise the possibility that for hemodialysis patients with anemia, Epo may be associated with increased formation of bone matrix [31]. Further supporting this notion, while Epo administration increased osteoclast numbers in marrow along the endosteal surfaces to facilitate the expansion of marrow, cortical thickness was enhanced [32].

How *in vivo* administration of Epo increases bone has yet to be determined. Our data show that the endogenous production of osteoblasts from mixed stromal populations and that BMPs produced by HSCs are likely candidates. At the same time, it is unclear whether HSCs and/or stromal cells or both are the cellular targets *in vivo*. One scenario worth considering is that Epo activation of HSCs results in the formation of osteoclasts which in the absence of nuclear factor kappaB-ligand (**RANKL**) and other overt inflammatory stimuli are unable to progress to fully activated osteoclasts capable of resorbing bone. The combined effects of either HSC-derived BMPs and Epo effects on stromal cells in the absence of activated osteoclast activity are likely to lead to a net gain in bone formation. The balance between osteoblast and osteoclast formation is crucial for bone formation. In the current study, we found that Epo stimulates osteoclastic activity and increases BMP-expressing HSC number in the marrow at 2 weeks of Epo treatment, while Epo enhances osteoblastic formation at 4 weeks (**Figure 6**). These data suggest that Epo not only regulates hematopoiesis but also participates in both osteoblastogenesis and osteoclastogenesis to form bone (**Figure 7A**). Moreover, these data further suggest that these events are coupled so that osteoclastic resorption occurs first and then bone formation follows (**Figure 7B**). It is also interesting to note that systemic Epo treatments increased the number of HSCs in the marrow of treated mice, but not by increasing marrow levels SDF-1/CXCL12, while PTH did. At the same time, systemic PTH did not result in increases in circulating Epo serum levels. Together these data suggest that bone formation by Epo and PTH may proceed along divergent pathways. PTH may work on osteoblastic precursors and perhaps mature blood elements, while the Epo pathway targets HSCs and osteoblastic precursors.

In summary, these data for the first time demonstrate that Epo regulates the formation of bone by both direct and indirect pathways, and further demonstrates the exquisite coupling between hematopoesis and osteopoiesis in the marrow. We have found that Epo/EpoR signaling in HSCs and/or stromal populations may lead to expansion of osteoclastic and osteoblast cell numbers. However, due to a predominant effect on osteopoiesis, a net gain in bone formation occurs. Although further study is warranted, these results suggest that targeting the Epo pathway may serve as a therapeutic modality to treat skeletal or mesenchymal abnormalities in humans.

## Materials and Methods

### Induction of Hematopoietic Stresses

4-to 6-week-old C57BL/6 mice (Charles River Laboratories, Wilmington, MA) were bled by jugular venipuncture under a protocol approved by the University of Michigan Committee for the Use and Care of Animals at the University of Michigan (Approval #8418), as previously describe [2]. The mice were anesthetized and approximately 20–30% of the calculated blood volume (∼0.55 ml for a 20 g mouse) was removed. Control mice were also anesthetized and subjected to puncture without hemorrhage.

### HSC Isolation

The bone marrow were flushed from the femur, tibia, and humeri with Dulbecco's Phosphate-Buffered Saline (D-PBS) (Invitrogen, Carlsbad, CA) without calcium and magnesium, supplemented with 2% heat-inactivated fetal bovine serum (**FBS**; Invitrogen). A single-cell suspension was obtained by gentle agitation through the syringe. Debris and remaining cellular aggregates were removed by passing the cell suspension over a 40 µm-mesh nylon cell strainer (BD Biosciences, San Diego, CA). Cells were incubated first with a biotinylated anti-Lineage (CD5, CD45R [B220], CD11b, Gr-1 [Ly- 6G/C], and Ter-119) antibody cocktail (Miltenyi Biotec, Auburn, CA) for 10 min at 4°C, then rinsed and stained with an antibody cocktail of APC-anti-stem cell antigen 1 (**Sca-1**) (clone D7; eBioscience, San Diego, CA), PE/Cy7-anti-c-Kit (clone 2B8; BioLegend, San Diego, CA), PE-anti-CD150 (clone TC15-12F12.2; BioLegend), FITC-anti-CD41 (clone MWReg30; BD Biosciences), FITC-anti-CD48 (clone BCM-1; BD Biosciences), and FITC- anti-Biotin antibodies (Miltenyi Biotec) for another 20 min at 4°C. HSCs isolated on the basis of the signaling lymphocyteactivation molecule (**SLAM**) family of receptors (**SLAM HSCs**) were sorted on a FACS Vantage dual laser flow cytometer (Becton Dickinson, San Jose, CA) by gating on cells that are Sca-1^+^c-Kit^+^CD150^+^CD41^−^CD48^−^Lin^−^, as previously described [2]. In some cases, Lin^−^Sca-1^+^c-Kit^+^ (**LSK**) cells were obtained using an antibody cocktail of APC-anti-Sca-1 antibody, PE/Cy7-anti-c-Kit antibody, and FITC-anti-Biotin antibody/biotinylated anti-Lineage antibody cocktail.

### Bone Marrow Cell Preparations

Bone marrow was flushed from the femurs, tibia, and humeri with Hank's buffer salt solution (Invitrogen). The cells were plated in α-MEM with 10% FBS and 1% penicillin and streptomycin. After 3 days, non-adherent cells were removed and fresh media were replaced.

### *In Vitro* Epo Treatments

SLAM HSCs, LSK cells, or bone marrow cells were incubated with rhEpo (5–10 U/ml) (EPOGEN; Amgen, Thousand oaks, CA) for 12 h. In some case, cells were also treated with Janus kinase 2 (**Jak2**)/Signal transducer and activator of transcription 3 (**Stat3**) inhibitor (AG490; Calbiochem, La Jolla, CA).

### Enzyme-Linked Immunosorbent Assays (ELISA)

Epo levels (R&D Systems, Minneapolis, MN) of the animal serum, Alkaline phosphatase (**ALP**) activity levels (Sigma-Aldrich, St. Louis, MO) of the cell lysates, and CXCL12 levels (R&D Systems) of the bone marrow supernatants were determined by double-antibody sandwich method assembled with commercially available components, according to the directions of the manufacturer. Data were normalized to the total protein.

### RNA Extraction and Real-Time RT-PCR

Total RNA was isolated using RNeasy Mini or Micro Kit (QIAGEN, Valencia, CA). First-strand cDNA was synthesized in a 20 µl reaction volume using 0.4 µg of total RNA or using MessageBooster cDNA synthesis kit when evaluating mRNA levels from isolated SLAM HSCs (Epicenter Biotechnologies, Madison, WI). RT products were analyzed by real-time RT-PCR in TaqMan® Gene Expression Assays of several target genes: mouse EpoR, BMP2, Runx2, osteocalcin (**OCN**), bone sialoprotein (**BSP**), Collagen I, and β-Actin (Applied Biosystems, Foster City, CA). Reactions without template and/or enzyme were used as negative controls. The 2^nd^ step PCR reaction were run for 40 cycles (95°C for 15 sec and 60°C 1 min) after an initial single cycle of 50°C for 2 min and 95°C for 10 min. The PCR product was detected as an increase in fluorescence using an ABI PRISM 7700 instrument (Applied Biosystems). RNA quantity (C_R_) was normalized to the housekeeping gene β-Actin control by using the formula C_R_ = 2^(40-Ct of sample)-(40-Ct of control)^. The threshold cycle (Ct) is the cycle at which a significant increase in fluorescence occurs.

### Flow Cytometry

LSK cells, SLAM HSCs, and bone marrow stromal cells (**BMSCs**) were stained with Alexa Fluor 488 anti-mouse monoclonal EpoR antibody (goat IgG; R&D systems) or IgG isotype-matched control after cell sorting. Anti-mouse EpoR antibody was stained with Alexa Fluor 488 monoclonal antibody labeling kit (Molecular Probes, Carlsbad, CA), according to the directions of the manufacturer. The levels of EpoR were analyzed by a FACS Vantage dual laser flow cytometer (Becton Dickinson).

### Western Blot Analyses

SDS-PAGE was performed in 10% polyacrylamide gels. After electrophoresis, the proteins were transferred to polyvinylidene difluoride membrane (Bio-Rad Laboratories, Hercules, CA). The membranes were then incubated with the primary antibody (The anti-EpoR antibody, R&D systems; the antibodies to phosphorylated Jak2 and Stat3, Cell Signaling Technology, Danvers, MA; the anti-BMP2 antibody, R&D systems; and the anti-Tubulin antibody, Sigma-Aldrich) overnight, and the secondary antibody (anti-species specific horseradish peroxidase, Cell Signaling Technology) was added. Finally the proteins were visualized by autoradiography using an enhanced chemiluminescence detection system (Amersham Pharmacia, Piscataway, NJ). The densities of the bands were quantified with Image J software (version 1.40; National Institutes of Health (**NIH**), Bethesda, MD).

### Mineralization Assays

BMSCs (2×10^5^ cells/well) were plated onto a 24-well culture plate with osteogenic media containing 50 µg/ml ascorbic acid (Sigma-Aldrich) and 10 mM β-glycerophosphate (Sigma-Aldrich). BMSCs were treated with either vehicle as negative control, rhBMP2 (200 ng/ml) (R&D Systems) as positive control, or rhEpo (5–10 U/ml). At 14 days, osteoblastogenesis from BMSCs were evaluated by Alizarin Red staining (Sigma-Aldrich).

### *In Vitro* Osteoclastogenesis

Marrow mononuclear cells (**MMCs**) (1×10^5^ cells/well) were plated onto 96-well culture plates. Cells were treated with rhRANKL (50 ng/ml) (R&D Systems) and/or rhEpo (5–10 U/ml) every other day for 7 days. Thereafter, osteoclastogenesis were evaluated by TRAP staining (Sigma-Aldrich).

### *In Vitro* Bone Resorption Assays

Bone resorption assays were performed as previously described [33]. MMCs (5×10^5^ cells/well) were seeded on the resorbable artificial bone film-coated disks (Becton Dickinson) and incubated with vehicle, rhEpo (10–20 U/ml), or rhRANKL (50 ng/ml). After 3 weeks of culture, the mean area of resorption from five randomly selected fields was analyzed.

### *In Vivo* Bone Formation Model

Newborn or 4-to 6-week-old C57BL/6 mice (Charles River Laboratories, Wilmington, MA) were treated with 50 µl of vehicle (0.9% saline) as negative control, parathyroid hormone (50 µg/kg, per day) (hPTH(1–34); Bachem, Torrence, CA) as positive control, or rhEpo (1500–6000 U/kg, three times per week) by intraperitoneal injection in for 28 days.

### Micro-computed Tomography Evaluations

Lumbar vertebrae were harvested at 28 days of treatment. For micro-computed tomography (**μCT**) analysis, specimens will be scanned at 8.93 µm voxel resolution on an EVS corp. μCT scanner (London, Ontario, Canada), with a total of 667 slices per scan. GEMS MicroView® software was used to make a 3-D reconstruction from the set of scans. A fixed threshold (1,000) was used to extract the mineralized bone phase, and bone mineral density (**BMD**), and bone volume fraction (**BVF**) were calculated.

### Immunohistochemistry

Seven µm sections of the decalcified long bones were then cut with a microtome (Leica RM2125 RT; Leica Microsystems, Bannockburn, IL) and stained with hematoxylin and eosin (**H&E**) and TRAP staining kit (Kamiya biomedical company, Seattle, WA). Total bone areas were measured using a computer-assisted bone histomorphometric analyzing system (Image-Pro Plus v.5.1; Media Cybernetics, Silver Spring, MD).

### Immunohisto-fluorescence Assays

Seven µm frozen sections were fixed in acetone at −20°C for 10 min and were blocked with Image-iT FX signal enhancer (Invitrogen) for 30 min. The slides were incubated overnight at 4°C with anti-CD150 monoclonal antibody (BD Biosciences) (pre-stained with Zenon® Alexa Fluor® 405 mouse IgG_1_ labeling kit; Invitrogen) and anti-BMP2 purified Goat IgG antibody (R&D Systems) (pre-stained with Zenon® Alexa Fluor® 555 Goat IgG labeling kit; Invitrogen). The next day, the slides were incubated for 4 h at 4°C with FITC-anti-mouse CD3e, Ly-76, CD45, CD41, and CD48 antibodies (eBioscience). Confocal laser scanning was performed with a FV500 confocal laser-scanning microscope (Olympus, Center Valley, PA). CD150^+^BMP2^+^CD41^−^CD48^−^Lin^−^ area was measured with Image Pro 5.1 program.

### Colony Formation Assays

BMSCs (1×10^6^ cells/well) that were isolated from vehicle-treated, PTH-treated, or Epo-treated 4-to 6-week-old C57BL/6 mice were plated onto a 6-well culture plate with osteogenic media containing 50 µg/ml ascorbic acid and 10 mM β-glycerophosphate for 21 days. For osteoblast colony-forming unit (**CFU-OB**) and fibroblast colony-forming unit (**CFU-F**) enumeration, the cultures were fixed with 10% normal buffered formalin, and stained with Alizarin Red and Crystal Violet (Sigma-Aldrich), respectively. Colonies with greater than 50 cells are counted.

### Statistical Analyses

Numerical data are expressed as mean ± standard error of the mean. Statistical analysis was performed by ANOVA or unpaired two-tailed Student's *t* test using the GraphPad Instat statistical program (GraphPad Software, San Diego, CA) with significance at *P*<0.05. For the real-time RT-PCR assays, a Kruskal-Wallis test and Dunn's multiple comparisons tests were utilized with the level of significance set at *P*<0.05.

## Supporting Information

Figure S1 Quantitative analysis of Western blots. The densities of the western blot bands were quantified with ImageJ software. (A) Quantitative analysis of (A) EpoR, (B) P-Jak2, and (C) P-Stat3 in HSCs (SLAM markers) that were treated with Epo with or without AG490 after 12 h in Figure 1D. Data are presented as the mean ± standard error of the mean from triplicate determinations. P: phosphorylated.
(0.21 MB TIF)
