# Supplementary figures and images for: Erythropoietin Couples Hematopoiesis with Bone Formation
Source: PLoS One. 2010 May 27;5(5):e10853. doi: 10.1371/journal.pone.0010853 (PMC2877712; doi:10.1371/journal.pone.0010853)

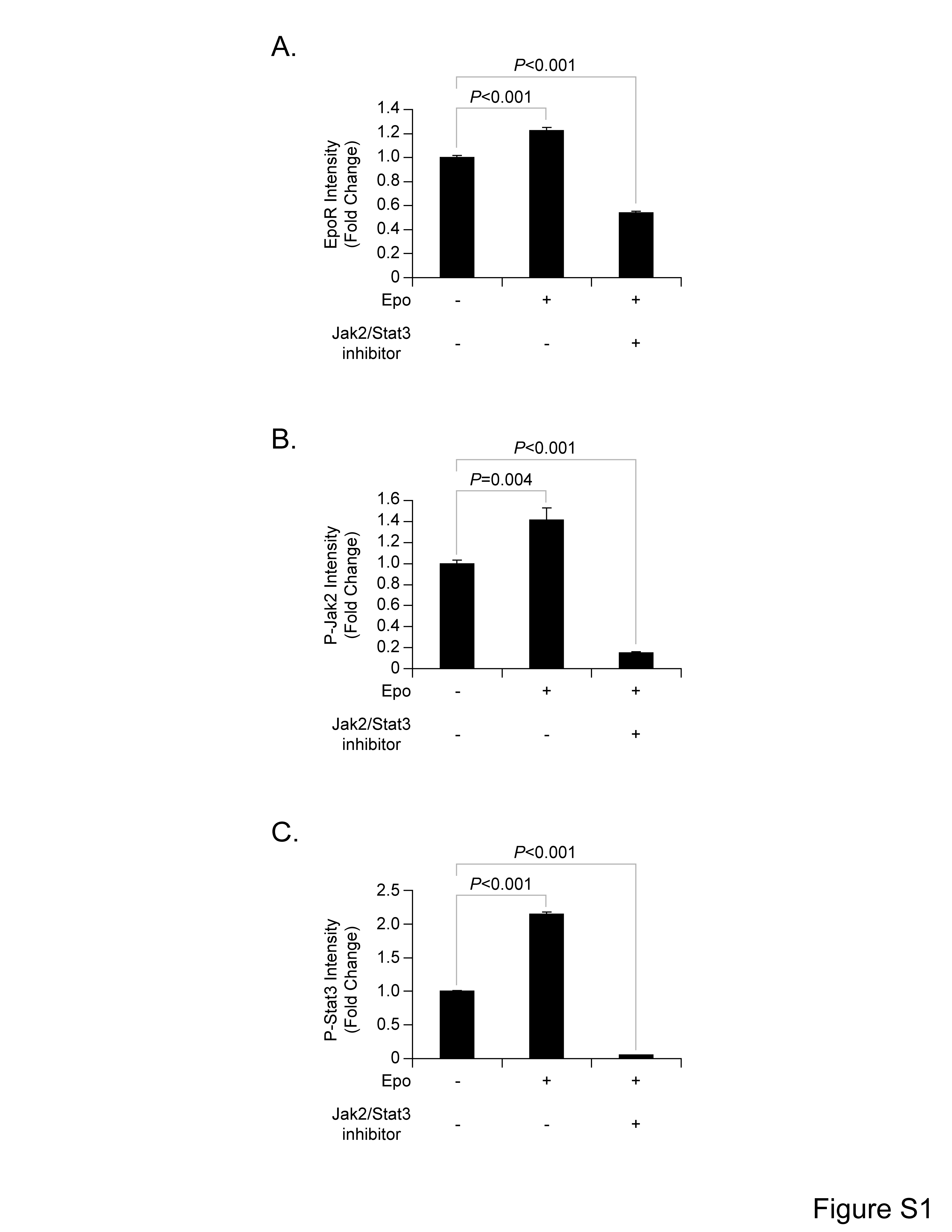

Supplement: Figure S1 — Quantitative analysis of Western blots. The densities of the western blot bands were quantified with ImageJ software. (A) Quantitative analysis of (A) EpoR, (B) P-Jak2, and (C) P-Stat3 in HSCs (SLAM markers) that were treated with Epo with or without AG490 after 12 h in Figure 1D. Data are presented as the mean ± standard error of the mean from triplicate determinations. P: phosphorylated. (0.21 MB TIF) [file pone.0010853.s001.tif]
